# Binding interface change and cryptic variation in the evolution of protein-protein interactions

**DOI:** 10.1186/s12862-016-0608-1

**Published:** 2016-02-18

**Authors:** Ryan M. Ames, David Talavera, Simon G. Williams, David L. Robertson, Simon C. Lovell

**Affiliations:** Computational and Evolutionary Biology, Faculty of Life Sciences, University of Manchester, Oxford Road, Manchester, M13 9PT UK; Current address: Wellcome Trust Centre for Biomedical Modelling and Analysis, University of Exeter, RILD Level 3, Exeter, EX2 5DW UK; Current address: Institute of Cardiovascular Sciences, Faculty of Medical and Human Sciences, University of Manchester, Oxford Road, Manchester, M13 9PT UK

**Keywords:** Protein-protein interactions, Gene duplication, Evolution, Protein complexes, Protein structure

## Abstract

**Background:**

Physical interactions between proteins are essential for almost all biological functions and systems. To understand the evolution of function it is therefore important to understand the evolution of molecular interactions. Of key importance is the evolution of binding specificity, the set of interactions made by a protein, since change in specificity can lead to “rewiring” of interaction networks. Unfortunately, the interfaces through which proteins interact are complex, typically containing many amino-acid residues that collectively must contribute to binding specificity as well as binding affinity, structural integrity of the interface and solubility in the unbound state.

**Results:**

In order to study the relationship between interface composition and binding specificity, we make use of paralogous pairs of yeast proteins. Immediately after duplication these paralogues will have identical sequences and protein products that make an identical set of interactions. As the sequences diverge, we can correlate amino-acid change in the interface with any change in the specificity of binding. We show that change in interface regions correlates only weakly with change in specificity, and many variants in interfaces are functionally equivalent. We show that many of the residue replacements within interfaces are silent with respect to their contribution to binding specificity.

**Conclusions:**

We conclude that such functionally-equivalent change has the potential to contribute to evolutionary plasticity in interfaces by creating cryptic variation, which in turn may provide the raw material for functional innovation and coevolution.

**Electronic supplementary material:**

The online version of this article (doi:10.1186/s12862-016-0608-1) contains supplementary material, which is available to authorized users.

## Background

Highly specific interactions between proteins are essential for almost all biological function [1, 2]. The full complement of interactions that make up all biological functions lead to complex interaction networks. These networks of protein-protein interactions (PPIs) that underpin biological function may arise either through gain of novel interactions [3], or by modification of the specificity of existing interactions. Here specificity is defined as the ability of a protein to physically interact with a specific set of other proteins to perform a function. When existing specificity is modified, it is frequently after a duplication event [4–7]. In order to understand the evolution of functions that arise from PPI networks, it is thus necessary to understand both duplication and the evolution of binding specificity.

Functional innovation has been inferred from accelerated rates of evolution within protein coding sequences [4, 8, 9]. However, changes in interaction specificity after duplication cannot be reliably identified from the evolutionary rate [10], probably because only a small fraction of residues are in binding interfaces. Even if only those residues in the interaction interface are considered [11], the relationship between substitutions and specificity change is complex. Residues within a binding interface may have a number of roles, including contribution to stability of the three-dimensional structure, interaction with solvent in the unbound state, and contribution to binding energy in addition to specificity [12]. Any of these factors have the potential to result in selection pressure on interface residues, complicating analysis of evolutionary change. Here, we aim to characterise the effect of interface change on physical interactions.

The availability of large-scale data sets for some organisms allows comparative analysis of PPIs and their evolution. In the yeast *Saccharomyces cerevisiae* a large number of duplicate genes have been identified, including a set that arose from whole genome duplication, and therefore are the same age [8, 13, 14]. In addition, many large scale interaction studies have extensively characterised the PPI network in yeast [15–17] and the three-dimensional structure is known for a substantial number of the protein complexes, allowing analysis of interactions and binding interfaces. Immediately subsequent to duplication the “daughter” genes will have identical sequences, and so will make the same sets of interactions. By comparing interaction specificity between duplicates we can estimate the frequency of network rewiring in these duplicate sets. By correlating these changes with substitutions in the interfaces, we aim to correlate interface substitutions with changes in specificity. The use of whole-genome duplicates also controls for the dating of duplication events, i.e. all the duplicates are the same age.

Here, we show that the rate of change in interfaces is only weakly correlated with the number of shared interactions between duplicates, indicating that there are a number of functionally equivalent substitutions (i.e., those that do not alter binding specificity) even within the binding interface. We identify specific substitutions in interfaces that are associated with the maintenance of a physical interaction. Our results demonstrate that specificity change can be partially understood by taking into account the specific structural context of individual interacting residues. In addition, we find that there is a large degree of variation in the interfaces that is both functionally equivalent and evolutionarily neutral (i.e., where variants are selectively equivalent). We suggest that interfaces exhibit cryptic variation [18], in that some variation does not contribute to phenotype in the context in which it is found, but in combination with further mutations can lead to functional change. In combination these diverse variation types allow evolutionary plasticity, offering a pathway to later functional innovation.

## Methods

### Genomic data

All open reading frames for *S. cerevisiae* were downloaded from the *Saccharomyces* Genome Database (SGD). Duplicate pairs were annotated using previously determined duplicate genes: 1) Whole genome duplicates were annotated using data from Kellis et al. [8]; 2) Small scale duplicates were annotated using data from Hakes et al. [14]. In total we have information for 720 duplicate pairs with mean identity of 62.05 % ± 21.78 %. The rate of nonsynonymous (K _*a*_) and synonymous (K _*s*_) substitutions were determined for each duplicate pair by aligning the protein sequences of duplicates using MUSCLE (v 3.8.31) with default parameters [19] and then converting these alignments to codon alignments using unaligned nucleotide sequences. K _*a*_ and K _*s*_ were then estimated using yn00 of the PAML (v 4.8) package with the universal genetic code, no weighting to count differences between codons and with common codon frequencies for all pairwise comparisons in the data [20]. A list of these duplicate genes along with details on duplication event and substitution rates can be found in Additional file 1: Table S1.

### Yeast complex structures and identifying interaction interfaces

To create a set of yeast protein complexes we followed the method of Talavera et al. [21]. Here, we obtained the structures of yeast proteins in complex from the PISA database [22], in each case selecting the most likely conformation while removing protein chains shorter than 50 residues and ligands from the analysis. The removal of short protein chains and ligands from the analysis aims to avoid including spurious interactions involving peptides or protein fragments that may be identified in high-throughput screens though the presence of these peptides cannot be ruled out. Removing these chains means that our analysis may focus much more on obligate interactions rather than transient physical interactions. We also used BLAST (v 2.2.17) [23] with default parameters to identify any PISA complexes containing orthologues to known yeast genes. Here, individual protein chains were extracted from the PISA structures and those with ≥80 % sequence identity to a BLAST-formatted yeast protein database were considered orthologous. On average the largest aligned region for these BLAST hits covered >70 % of the yeast protein sequence indicating high global alignment identity of orthologues.

To generate yeast versions of these protein complexes we used Modeller (v9.11 Sept 2012) [24] with default parameters to model yeast specific versions. These modelled complexes were assembled using FatCat (via biojava v 3.0) [25] with flexible superimposition and hydrogen atoms were added to the structures using Reduce (v 3.23) with default parameters [26]. All structures were included in this analysis regardless of resolution to maximise the data available. Although this means that some of the models used may contain some degree of error, the median resolution of all the template structures used in this analysis is 2.3 Å, and only 16 and 8 % of structures have a resolution worse than 2.8 Å and 3 Å, respectively.

We selected those structures where at least one member of the complex had a known paralogue giving us an initial data set of 166 structures, some of which contained multiple paralogoues. We note that some duplicates may be represented more than once in our set if they were found in more than one complex or to interact with more than one protein in a single complex. For all of these structures we generated a model of the paralogue using Modeller. For each modeller run we generated 10 models of the paralogue using the sister protein in the known complex as the template. The discrete optimised protein energy (DOPE) score was used to assess the accuracy of the models and the model with the lowest DOPE score was used in further analysis. Hydrogen atoms were added with Reduce and the best model was superimposed on its sister protein using FatCat. At this stage we have yeast proteins in complex from the PISA database with a modelled paralogous structure superimposed. This allows us to identify the interaction interfaces in both duplicate genes and identify any substitutions present in these interfaces.

Interaction interfaces were identified using Probe (v 2.16) to detect interactions between the source and target as well as the target and source [27]. Residues are defined as being in contact by Probe if the distance between a single pair of atoms in the two chains is less than the sum of their van der Waals radii + 0.5Å. All contacting residues between two chains were defined as the interface between those chains. Any contacts that were described as clashes or bad overlaps by Probe were also included as interface residues to account for any side chain positioning errors introduced by Modeller and FatCat. In order to assess the goodness-of-fit of the interfaces in modelled structures we used the distribution of Probe contact scores across all interface regions. We find that on average all interface regions have an average Probe score (normalised by the number of contacts) of −0.064±0.033 measured from 154 PISA structures containing known duplicates with a successfully modelled paralogue (11 structures containing duplicates could not be modelled - see below). Interfaces of duplicates in known structures from PISA have an average Probe score of −0.060±0.033, whereas the modelled paralogues have an average probe score of −0.066±0.033. These values indicate that there is very little difference in goodness-of-fit between interfaces taken from PISA and the interfaces of modelled paralogues, suggesting that our modelling procedure has not introduced significant error.

We note that our definition of interface residues will include both residues that make contacts through side-chain interactions and residues with buried side-chains that make contacts through the main-chain. Examining the solvent accessible surface area (SASA) of interface residues in this study using POPS [28] reveals that on average interface residues expose ∼20 % of their surface area. Using a definition of ≤5 % SASA to classify a residue as buried [21], we find that <8 % of interface residues are buried and may interact through main-chain interactions. Thus, our analysis predominately focuses on interface residues that are located in the surface of a protein.

From the full PISA data set we identified 777 unique interaction interfaces of proteins participating in homo interactions and 189 interfaces participating in hetero interactions. Of these a total of 181 interfaces, 154 in homo interactions and 27 in hetero interactions, involved duplicates. After duplicate structure modelling, super-imposition, and attempting to identify the corresponding interaction interfaces in the duplicates we were left with a total of 170 interfaces with 74 (43.6 %) containing small scale duplicates and 96 (56.4 %) containing whole-genome duplicates. For 11 structures there were no residues structurally equivalent to the interaction interface in the duplicate protein, and so these complexes were omitted. Previous researchers have divided complexes into “transient” and “obligate” interactions [29]. Such a division is not possible for our data set because the binding affinity is unknown for the majority of the interactions, and there is only a small overlap between our duplicate pairs and those where the interaction status is known [29].

### Interactions and shared interaction ratio

Physical interactions were assigned using the BioGrid (v 3.0) database [30]. BioGrid contains qualitative information on interactions i.e., whether or not two proteins interact, rather than quantitative data on binding affinity. An alteration in binding specificity is identified based on the comparison between the ability to form an interaction to the target ligand versus the ability to bind other targets, and so specificity changes are identified if a member of a duplicate pair either gains or loses an interaction.

For analysis we used a multiple confidence network based on multiple evidence from BioGrid and referred to as the MC network. Here, an interaction is only included in the network if it has been identified by two different studies. We note that in this definition interactions identified in multiple high-throughput studies will be included in our multiple confidence network suggesting that there is still the potential for this network to contain false positives. However, the inclusion of high-throughput studies with different methodologies may limit this bias. Additionally, small-scale studies also contain bias towards well-studied proteins. Importantly, previous work examining yeast protein interactions in mediating essentiality has found consistent results from networks built with a variety of confidence limits and thresholds [2]. We also carried out a subset of our analyses using all physical interactions listed in BioGrid to check the robustness of our results.

Interactions from PISA were included if they were not listed in BioGrid. This ensures that any protein with an identified interaction interface from a structure is listed as having at least one interaction. In the MC network we have a total of 11,847 interactions from BioGrid and 663 (5.5 %) additional interactions from PISA. Where we use all interactions listed in BioGrid we have a total of 53,573 interactions directly from BioGrid and a further 486 (0.9 %) from PISA.

The shared interaction ratio [10] (SIR) was calculated for all duplicate pairs, in which both members have at least one interaction, according to:

$$ SIR=\frac{s \times 2}{n_{1} + n_{2}} $$

where *SIR* is the shared interaction ratio, *s* is the number of interactions shared between the duplicates, and *n*_1_ and *n*_2_ are the number of interactions for duplicate A and B of the duplicate pair respectively.

### Frequency of specificity change

We use the method of Wagner [31] to calculate the frequency of specificity change. We calculate the rate of interaction loss by identifying the changes in the ancestral set of interactions for a set of duplicate genes. We infer the ancestral interaction sets of these genes by assuming each gene in a duplicate pair contained the same set of interactions immediately after duplication. For example, consider duplicate genes *A* and *B*. If *A* interacts with *C* then we infer that immediately after duplication both *A* and *B* interacted with *C*. We then calculate the frequency of specificity change as:

$$rate=\left(\frac{l}{a}\right)\left(\frac{1}{d}\right) $$

where *rate* is the rate of interaction loss per PPI per million years, *l* is the number of interactions that have been lost since duplication, *a* is the ancestral number of interactions immediately after duplication and *d* is the time in million years since duplication. In order to use an accurate estimate of divergence time we performed this analysis on whole genome duplicates, which are approximately 100 million years old [13].

Notably, this method makes the assumption that all divergence that has occurred in interactions has occurred from loss of interactions. It is likely that gain of interactions has contributed to the current interaction profiles of these genes though the method used here is unable to account for these gains. Additionally, we also cannot detect interaction loss events that have occurred in both members of a duplicate pair.

### Rate of change in interaction interfaces

To calculate the rates of substitution in interfaces between paralogues we used the protein sequences with identified interface residues and identified the corresponding nucleotides in the coding sequences. These highly similar duplicate sequences were aligned using MUSCLE with default parameters [19]. Substitutions between the duplicates both within and outside interfaces were identified. Non-interface regions were simply defined as any region that was not in an identified interface and will consist of both core and surface residues. We used codeml of the PAML package [20] to determine the substitution rate within interfaces relative to the substitution rate outside the interfaces. Codeml was run to analyse amino acids with a user defined tree (containing only the duplicate pair) and the WAG substitution model. All other parameters were left to default setting as described in the PAML manual. Any duplicates that showed more than twice the rate of change in interfaces compared to non-interfaces were removed from the analysis in order to exclude erroneous estimates of substitution rates. The result of this analysis is a relative interface substitution rate.

### Identifying selection in duplicate genes

To identify residues under selection we first created a multiple sequence alignment for each duplicate pair. The alignments were constructed from the duplicates and their closest orthologue from 8 species of yeast. The sequences for these species (*Eremothecium gossypii, Candidia glabrata, Debaryomyces hansenii var. hansenii, Kluyveromyces lactis var. lactis, Kluyveromyces thermotolerans, Saccharomyces kluyveri, Yarrowia lipolytica* and *Zygosaccharomyces rouxii*) were obtained from the Génolevures project [32]. In order to identify orthologues of duplicates in the other yeast species we used BLAST with default parameters [23]. Each duplicate protein was used as a query to search each yeast proteome and the top hit was selected as the closest orthologue in that species. Of these top hits 36–47 % are reciprocal best BLAST hits between species. Single directional hits were included to ensure as many ortholougues sequences as possible were included in the analysis. On average 57.7 % of genes that occur in an orthologue group in this study also occur in a single Génolevures protein family [33] indicating a substantial overlap with an existing set of orthologues identified from clustering of protein families. Multiple sequence alignments were constructed from the protein sequences of the duplicate genes and their orthologues from all 8 yeast species using MUSCLE [19] with default parameters. Codon alignments were generated using the aligned proteins and unaligned coding sequences.

Phylogenetic trees were inferred from the multiple sequence alignments using RAxML (v 7.2.6) [34] with the WAG +*Γ* model with 10 bootstraps and a full maximum likelihood search to identify the most likely tree. Any polytomies identified in these trees were resolved using the APE package in R [35]. The single-likelihood ancestor counting (SLAC) method of the HyPhy package (v 2.13) [36] was used to infer residues under selection in the codon alignments, using the best tree inferred by RAxML as the underlying phylogeny. In the SLAC method, nucleotide and codon model parameter estimates are used to reconstruct ancestral codon sequences at the internal nodes of the tree. The single-most likely ancestral sequences are then fixed as known variables and applied to inferring the expected number of nonsynonymous or synonymous substitutions that have occurred along each branch, for each position. Using HyPhy we estimated the K _*a*_ and K _*s*_ for each alignment column with branch correction and used a cutoff of *P*<0.05 to identify significantly positively and negatively selected sites. Variants with no significant selection signal were inferred to be neutrally evolving.

To determine whether there were significant differences in selection in interfaces where an interaction is conserved or diverged we made several comparisons of sites under negative selection and evolving neutrally. Furthermore we made comparisons between interface and non-interface residues in duplicates with both conserved and diverged interactions, although we did not make any comparisons to positively selected sites as very few positively selected sites were identified. To determine statistical significance of these comparisons we used the Kruskal-Wallis test with repeated Mann-Whitney tests for individual comparisons. To correct for multiple testing we used the method of Benjamini and Hochberg [37].

## Results and discussion

### Changes in interfaces and the evolutionary consequences for the protein-protein interaction network

**Fig. 1:**
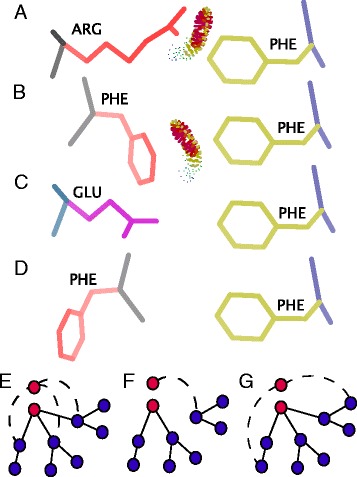
Potential interface substitutions that may maintain or alter a physical interaction and the evolutionary consequences for the protein-protein interaction network. In this hypothetical case an arginine (ARG) makes a physical interaction with a phenylalanine (PHE) at a binding interface enabling a physical interaction (**a**). The substitution of an ARG to a PHE may preserve the physical interaction if the PHE can still contact the interacting residue across the binding interface (**b**). However substitutions to amino acids that can no longer contact the interacting residue (or chain) because they cannot bridge the interface (**c**) or are orientated away from the interface (**d**) may result in an alteration of the physical interaction. In **a**–**d** protein backbones are shown in grey and blue with residue side-chain in red, purple and yellow. Yellow and red dots indicate a physical contact between residues. Changes in physical interactions have several potential consequences for the protein-protein interaction network. If we consider the case of a duplication event (red nodes in **e**, **f** and **g**), immediately after duplication the duplicate interfaces will be identical and all physical interactions will be maintained (**e**). Selection may favour this scenario (dosage benefit) and changes in interfaces may be constrained to maintain the interactions. Alternatively, substitutions in interfaces may lead to a divergence of interactions between the duplicates (subfunctionalisation - **f**). It is also possible, in rare cases, that divergence in binding interfaces may result in the formation of novel physical interactions (neofunctionalisation - **g**). In the network diagrams blue nodes represent proteins, red nodes duplicate proteins and edges between nodes depict physical interactions

Changes in binding interfaces may either disrupt a physical interaction or be functionally equivalent and maintain a physical interaction. There are many types of changes, e.g. changes that preserve side-chain interactions between residues in interfaces, that may explain the maintenance or divergence of an interaction and some potential scenarios are outlined in Fig. 1. Divergence of physical interactions has consequences for the evolution of the protein-protein interaction network.

In this study we examine duplicates in order to investigate the evolution of protein interactions. Duplicates with identical interaction interfaces or containing only functionally equivalent substitutions, will maintain all physical interactions. After duplication we might expect selection to favour maintenance of interactions if there is a selective advantage for increased dosage of the protein. Alternatively, changes that affect interactions may cause a partitioning in interactions between the duplicates, a process referred to as subfunctionalisation. Finally, interfaces may diverge such that novel interactions are formed, referred to as neofunctionalisation (Fig. 1).

### Duplicate genes are distributed across the protein-protein interaction network but represent mostly homomeric interactions

**Fig. 2:**
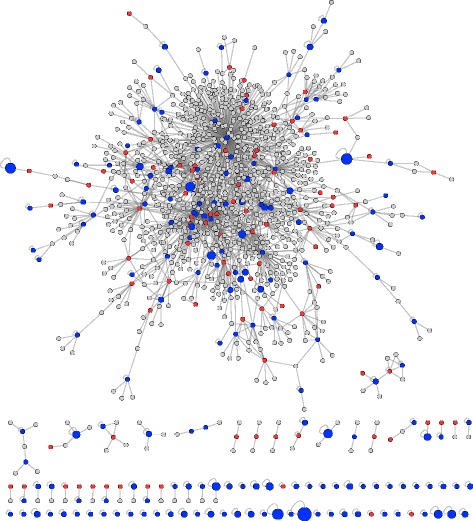
A network visualisation of duplicate genes and their known interactions. Duplicate genes with known interfaces from the PISA database are shown in blue. Duplicate genes with no known interfaces are shown in red. Singleton genes that interact with duplicates are shown in grey. Physical interactions between the protein products of these genes were extracted from the PISA [22] and BioGrid [30] databases and form the edges in the network. The size of the blue nodes is scaled with the number of known interfaces identified in the PISA database with larger nodes containing a larger number of known interfaces

A network visualisation of the interactions of duplicate genes shows that a large number of duplicates are identified as having homomeric interactions in the PISA database (Fig. 2). Interestingly, some duplicate genes that are only known to self-interact are identified as having several interfaces (large blue nodes). Single proteins can form homomeric complexes with more than one symmetry type and so use multiple interaction interfaces to bind several subunits [38]. Approximately 85 % of known interfaces in duplicate genes are homomeric so the conclusions drawn from this study will mostly describe the evolution of homomeric interactions.

Of the 1152 proteins in the network participating in 1852 interactions, 9 proteins have >40 interactions each, accounting for a total of 745 interactions. These proteins, which are all the products of duplicate genes, can be classed as network hubs. Hub proteins in this network are chaperone proteins (SSE1 and HSP82) or histones involved in chromatin assembly or chromosome function (HHF1, HHT1, HHT2 and HTA2) and therefore, are highly important for cell viability. The removal of these hubs has the potential to fragment the network and may result in the disruption of specific functions. Indeed hub proteins have been previously associated with essential genes [2, 39, 40], have been linked to duplicate genes with compensated functions [41] and have a high rate of interaction turnover [6].

The presence of hubs in our analysis has the potential to skew our analysis such that our results may only be applicable to the evolution of highly connected proteins. The blue nodes in Fig. 2 represent duplicates with known interactions and structures in the PISA database; effectively these nodes represent the proteins analysed in this study. We can see that these proteins occur at both the centre of the network where they act as hubs and at the periphery of the network where they have few interactions. Furthermore, the size of the blue nodes represents the number of interfaces identified in these proteins and the presence of peripheral proteins with many interfaces indicates that our analysis has not disproportionately focused on hubs. Therefore, despite the presence of hub proteins in our analysis, our data describe the evolution of proteins with both many and few interactions.

### Divergence of protein-protein interactions between duplicate genes occurs more rapidly than previously thought

We have determined the rate of divergence of interactions after duplication. Previous estimates range from 10 ^−6^/PPI/Myr to 2.3 ×10^−3^/PPI/Myr [31, 42–44]. This variability of estimates is due to some methods estimating only the rate of interaction loss, while others estimate the rate of both gains and losses. Additionally, the technical difficulty of estimating the time since duplication also confounds estimates of interaction divergence. Here, we estimate the rate of interaction loss, which is comparable with the rate estimated by Wagner [31]. We assume that differences seen in interactions are predominantly caused by interaction loss and we cannot account for losses that cannot be observed *i.e.* those interactions that have been lost by both duplicates. Finally, we estimate the rate of interaction loss using only those duplicates generated by the whole-genome duplication to control for duplicate age. These genes all duplicated simultaneously, and the event has been dated, on the basis of the 18S RNA molecular clock and the estimate of the animal/fungi divergence, at around 100 million years ago [13].

**Fig. 3:**
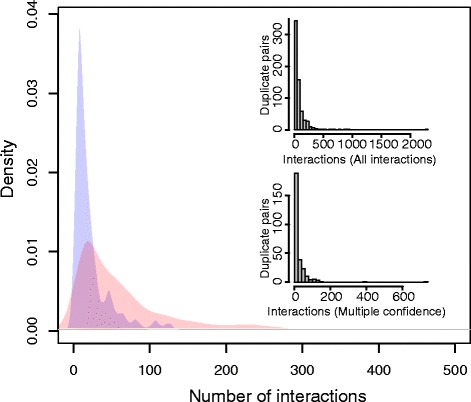
Density and frequency distributions of the total number of interactions observed per duplicate pair. In the density distribution the blue curve represents the density of interactions from all interactions contained in BioGrid. The red curve shows the density of interactions from the multiple confidence network (interactions must be observed in two separate studies). The density distribution describes the probability of a duplicate pair having a specific number of interactions and the area of each curve sums to 1. The insets show the frequency distributions of interactions per duplicate pair with interactions from all interactions contained in BioGrid (*top*) and interactions from the multiple confidence interaction network (*bottom*)

From a total of 204 whole genome duplicates with interaction data in the MC network (interactions must be observed in multiple studies in BioGrid [30]), we calculate the rate of interaction loss to be 6.0 ×10^−3^/PPI/Myr. When using all interactions we are able to analyse 420 whole genome duplicates and estimate the rate of interaction loss to be 5.8 ×10^−3^/PPI/Myr. The density and frequency distributions of interactions per duplicate are shown in Fig. 3. We can see that the distributions of interactions are different for the two networks, which likely reflects the presence of missing interactions and false positives in the MC and all interaction networks respectively. Despite the differences in interaction distribution we find that the estimated rates of interaction loss are similar for both networks, suggesting that our estimate is robust to the choice of network. These estimated rates are higher than any produced previously, particularly the rate of interaction loss (10 ^−6^/PPI/Myr) estimated by Wagner [31].

Wagner [31] discussed the problem of estimating the age of duplication events, and suggested that this uncertainty may lead to an artificially low estimate of interaction loss. We did not test the rate of interaction loss in small-scale duplications because of uncertainties in the age of the duplication events. However, we might expect the rate of loss for small-scale duplicates to be greater than that for whole-genome duplicates [14, 45] because genes generated in the whole-genome duplication might be more likely to be dosage balanced than small-scale duplicates as the whole-genome duplication can encompass entire complexes. Dosage balanced genes may therefore be less likely to diverge in interactions as these changes could cause an imbalance. Nevertheless, we find a high rate of interaction loss in duplicate genes arising from the whole-genome duplication suggesting that interactions may diverge more rapidly after duplication than previously thought.

### Divergence of interactions does not correlate with substitutions along the whole protein sequence

If changes in protein coding regions lead to changes in interaction specificity, we would expect a negative correlation between the rate of change in proteins and their sets of interactions. Indeed, where it is known that interologs exist (i.e., conserved interactions that have interacting homologs in another organism, [46]), sequence similarity is predictive of whether the interaction mode is conserved across orthologues [47]. However, using the shared interaction ratio of paralogues to express changes in binding between duplicates, we find that there is no correlation between substitutions in the full protein sequence and SIR (*R* =0.044, *P* =0.43 for the MC network, *R* =0.089, *P* =0.12 for all interactions).

Hakes et al. [10] have previously shown that there is no correlation between substitutions in the protein sequence and SIR. These authors hypothesised that only a subset of residues, those in the interfaces, are responsible for maintaining an interaction and divergence in the rest of the coding sequence will mask these specific changes. Previous studies examining the effects of substitutions in human genes have shown that disease causing mutations occur preferentially in known interfaces rather than elsewhere on the protein surface [48, 49]. Analysis of evolutionary change in binding interfaces can indicate whether sequence changes are compatible with a maintained interaction [50], and this method has been used for large-scale assembly of protein complexes, when combined with electron microscopy and TAP-tagging data [51]. As such we focus the rest of our analyses on interaction interfaces.

### Interfaces between duplicate genes show signs of divergence

We next looked to identify changes in interfaces and used the set of paralogues to determine the rate of interface divergence. PAML [20] was used to determine the substitution rates in interfaces relative to non interface regions (i.e. regions not classed as being in an interface) between duplicate pairs. Here both surface and core regions of a protein are included as non-interface sites. As the core of a protein tends to be more conserved we are likely decreasing the observed rate of divergence in non-interface regions. Regardless, we see that the majority of duplicate pairs show a lower substitution rate for interfaces compared to non-interface regions; median substitution rate for interfaces relative to non-interface residues is 0.76 (Wilcoxon test P = 3.84 ×10^−12^). These results suggest that interface regions are under greater selective constraint than non-interface regions, which has been seen for other sets of PPIs [29]. However, many interface regions show signs of divergence (relative evolutionary rates of change >0), indicating that many duplicates show variation in their interface regions.

### Correlation of changes in interfaces with changes of interactions suggests many interface changes are functionally equivalent

The majority of duplicate pairs show some divergence in interface regions, and these duplicates also often differ in their interactions. We define diverged interfaces as those that contain one or more substitutions in any identified interface between paralogues. Diverged interactions are those that do not share all physical interactions in BioGrid [30]. The relationship between the two types of divergence is detailed in Table 1. We can identify more duplicate pairs that show a diverged interface with maintained interactions than duplicates that show conservation of both interface and interactions. This suggests that not all substitutions within interfaces have an effect on binding and that at least some substitutions within interfaces are functionally equivalent (for example see Fig. 1b).

**Table 1:** Relationship between change in interfaces and change in specificity, using the multiple confidence network. Values based on all interactions in BioGrid are shown in parentheses

		Interface	
		Conserved	Diverged
Interaction	Conserved	18 (18)	34 (56)
	Diverged	12 (12)	106 (84)

Although this analysis compares all identified interfaces with all known physical interactions, these proteins may contain additional interfaces that are not detected because no 3D structure exists. Likewise the set of all known physical interactions may contain both false negatives and false positives [52]. Indeed, we identify 12 duplicate pairs with conserved interfaces and diverged physical interactions. Alternatively, interaction changes may be caused by substitutions away from the interface that may, for example, affect protein folding, which may in turn affect the interface and binding specificity. Protein cores tend to be populated by hydrophobic residues and substitutions that alter the hydrophobic area, introduce charge or change the packing by introducing residues with different sizes may affect protein folding and function [53, 54].

If all or any changes in interfaces lead to changes in interaction specificity, we would expect a negative correlation between the rate of change in interfaces and SIR. However, we would expect this correlation to be weak if at least some interface substitutions are functionally equivalent. We observe a weak but significant negative relationship between interface substitution rate and SIR (Additional file 2: Figure S1). This is the case whether we use the MC network (*R* =0.170, *P* =0.01) or all interactions (*R* =0.236, *P* =0.001). A reduction in the number of shared interactions between duplicates coupled with diverging interface regions over evolutionary time may indicate a role for subfunctionalisation in the divergence of these duplicates. In this process it is expected that duplicates are losing complementary interactions such that both duplicates are required to perform all the ancestral interactions (for example see Fig. 1f). Importantly, the correlation between interface divergence and SIR is extremely weak. Coupled with the observation that changes in interfaces do not always alter specificity (Table 1), our results suggest that there are many interface substitutions that do not contribute to binding and specificity.

### Analysis of selection in interfaces reveals a prevalence of neutrally evolving sites

**Fig. 4:**
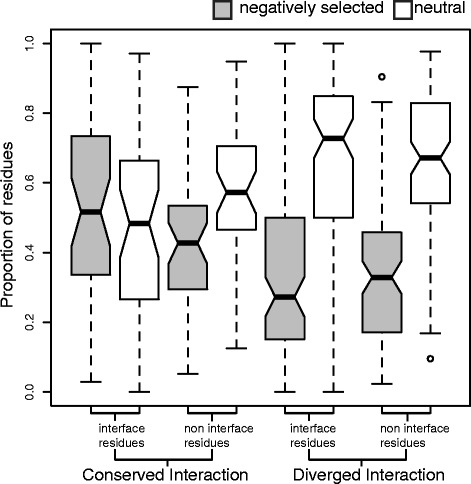
The difference in selection on interface and non-interface residues in duplicates with conserved and diverged interactions. The Boxplots show the proportion of residues in interfaces and non-interface regions under negative selection or neutrally evolving for duplicate pairs. Duplicate pairs showing conserved interactions at the interface have been separated from those that show diverged interactions. Categories of interface and non-interface residues are shown on the x axis. Boxplots for negatively selected residues are shown in grey and neutrally evolving residues plots are shown in white

Having demonstrated the presence of functionally equivalent substitutions in interfaces we next determined whether these interface residues were under selection. We hypothesised that interfaces with conserved interactions would show predominantly negative selection, and interfaces in duplicates with diverged interactions would show signs of high rates of change, indicating reduced purifying selection or even positive selection. HyPhy [36] was used to infer negative, neutral and positive selection at each position in multiple sequence alignments containing the duplicate pairs and orthologues from a range of other yeast species. We compared negatively selected or neutrally evolving sites across interfaces and non-interfaces, as well as between duplicates with conserved interactions and diverged interactions (Fig. 4). Sites with positively selected variants were not included in this analysis as very few were identified and they were always outside of binding interfaces.

A significantly higher proportion of sites in interfaces are under purifying (negative) selection when an interaction is conserved, compared to when the interaction is diverged (corrected Mann-Whitney *P*<0.05). In non-interface regions there are significantly more neutral substitutions than negatively selected substitutions regardless of whether the interaction has been maintained between the duplicates (corrected Mann-Whitney *P*<0.05). Interestingly, we see no significant difference between the proportion of interface residues displaying negative selection compared to those with neutrally evolving variants when the duplicates maintain an interaction. However, there is a significantly higher proportion of interface sites with neutrally evolving variants compared to those negatively selected when the interaction has diverged (corrected Mann-Whitney *P*<0.05). Overall, interfaces are constrained when compared with non-interface regions, and there is stronger constraint in cases where the interactions are conserved between paralogues.

A potential confounding factor in this analysis is that interfaces may be used for more than one interaction. After duplication and divergence, each daughter gene may retain a non-overlapping subset of these interactions (subfunctionalisation see Fig. 1f). Duplicates that have undergone subfunctionalisation will have diverged interactions, yet the interface may be under negative selection in order to maintain the partitioned interactions. In addition, results may be affected by the completeness and accuracy of the interaction data. Overall, the degree of negative selection found in an interface may not be indicative of a maintained or diverged interaction. Although interfaces are likely to be under negative selection to maintain some physical characteristics of the binding site, it may only be a subset of these residues that affects specificity.

The observation that a large proportion of residues are evolving neutrally (i.e., with no evidence of selection) and appear to be functionally equivalent (i.e. can be changed without affecting binding), even within binding interfaces, suggests potential evolutionary pathways that can lead to evolutionary change in complex systems such as signalling pathways and molecular machines. We identify a significantly higher proportion of residues in interfaces evolving neutrally when the interface is involved in a diverged interaction. Moreover, in interfaces involved in conserved interactions there are almost as many neutrally evolving residues as those under purifying selection (Fig. 4). These neutral changes may not alter binding specificity but may still affect binding affinity and kinetics [55]. Such changes may be described as cryptic or standing variation, defined as variation that does not contribute to the normal range of phenotypes observed in a population but in a different context (i.e. in combination with subsequent mutations) leads to functional change [18]. For example, with regard to PPIs, cryptic variation may allow duplicate pairs to follow different evolutionary pathways whereby an initial, functionally equivalent, change in one paralogue does not remove an interaction but allows subsequent specificity changing substitutions at other sites within the interface. Thus, these neutral changes in a new context may allow for subsequent neo- and sub-functionalisation. In addition, coevolution between interacting proteins is more likely in systems that are not highly constrained, and neutral change in interfaces may relax the constraint on change within the interface. We suggest, therefore, that the presence of functionally equivalent changes in interfaces is key for functional evolution and innovation in PPIs.

We are unable to find any signs of positive selection acting at binding interfaces. This might add further evidence for the role of neutral processes in the evolution of duplicate genes and their physical interactions. Duplicate genes can diverge in their physical interactions by subfunctionalisation [56] where loss of interaction mutations can occur by neutral processes. However, there are other possible explanations for the lack of positive selection signal in our data. There is substantial evidence that suggests that changes in protein sequences occur rapidly in one paralogue after duplication [57–59]. If residues undergo positive selection during this rapid divergence to alter their interactions and subsequently experience negative selection after this divergence, it may be difficult to detect any signal for positive selection. We might not expect this to affect our analysis as our selection inference method constructs ancestral sequences and looks for selection along all branches. Additionally, as we expect that only the minority of residues within an interface determine binding specificity, it may be very difficult to accurately identify positive selection at such a small number of sites. Nozawa et al. [60] have shown that some methods are unreliable at accurately identifying positive selection when the number of substitutions is low.

### Specific interface substitutions may maintain or alter interaction specificity

**Fig. 5:**
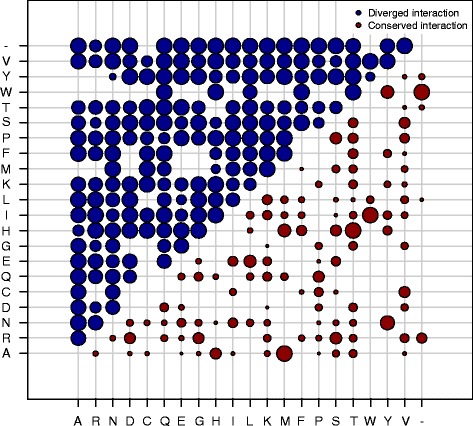
The proportions of substitutions in interfaces between duplicate pairs where there is a conservation or divergence in physical interaction using the multiple confidence interaction set. The area of each circle represents the frequency of the substitution. Red circles show those substitutions that are present between duplicate interfaces where the interaction is conserved. Blue circles represent substitutions between duplicate interfaces where the interaction has diverged. As we do not have the ancestral sequence before duplication all substitutions are treated as undirected i.e. A to R is the same as R to A

Our finding that many interface substitutions are functionally equivalent and/or neutrally evolving allows us to identify specific substitutions which maintain or change specificity. Figure 5 shows a substitution matrix where the colour of the circle shows whether the substitution is present between duplicates that have maintained or altered interaction specificity. The size of each circle represents the proportion of a particular substitution that occurs in an interface with a diverged or maintained interaction. We see that the majority of substitutions in interfaces lead to a divergence in interactions. Interestingly, we see that gaps in interfaces almost always lead to a change in interaction even though indels in interfaces are very rare in our duplicate set (<1 % of interface residues are gaps in duplicate alignments).

Duplicates with conserved interactions show fewer substitutions in their interface regions. With regard to specific amino acid types, met ⇔ala, thr ⇔his and trp ⇔ile substitutions are prevalent in conserved interactions. These residues have been shown to contribute significantly to binding energy and are frequently found in binding hot spots [55, 61–63]. This result shows that even substitutions of residues that are important for binding may be tolerated, dependent on structural context. Although conservative changes might maintain binding energy, residues important for binding energy may be distinct from those that determine binding specificity [12].

**Fig. 6:**
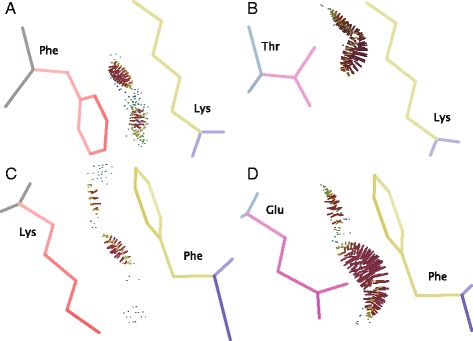
Substitutions in interfaces of RNR4 and RNR2 that maintain an interaction with RNR4. Substitutions are shown in consecutive panels with RNR4 coloured gray with red side chains and the duplicate RNR2 shown in light blue with purple side chains. The interacting chains (in this case also RNR4) are shown in blue with yellow side chains. Coloured dots between chains represent atom contacts between the proteins detected by Probe. A substitution between a phenylalanine (Phe) on RNR4 (**a**) and a threonine (Thr) on RNR2 (**b**) maintains atom contacts with a lysine (Lys) on the interacting chain. A second substitution between a lysine (Lys) on RNR4 (**c**) and glutamic acid (Glu) on RNR2 (**d**) maintains atom contacts with a phenylalanine (Phe)

Previous work focused on the evolvability of yeast interaction interfaces proposed that the structural context, including the types of atoms involved in interactions between protein chains, can affect a residues propensity to be replaced [64]. For example interface residues with small side chains or whose side chains are orientated towards the protein core will likely make interactions through their main-chain atoms, and thus, may be more likely to be replaced as any residue can make these types of interactions. Indeed, residues that make main chain interactions are less likely to be structurally constrained [64]. However, the majority (60.6 %) of interacting atoms found in interfaces are “residue-type specific”, i.e., neither main chain atoms, *β*-carbon nor *β*-hydrogens. Therefore, in some cases at least, interface substitutions that maintain an interaction must do so through side-chain interactions. Where side chains are orientated towards the interface and contact the interacting protein a substituted residue may still make the same atom contacts, based on its orientation and side chain, allowing the maintenance of the interaction (see Fig. 1b). For example the duplicate pair RNR4 and RNR2 are both able to interact with RNR4 despite two substitutions in the interaction interface. The changes between a phenylalanine and a threonine as well as a change between a lysine and glutamic acid preserve atom contacts with residues on the interacting protein and may be important in maintaining the interactions between these proteins (Fig. 6).

If we define interface substitutions that maintain atom contacts across an interface as substitutions that will maintain an interaction (Fig. 1a & b and Fig. 6) and any substitutions that do not maintain atom contacts as substitutions that will alter an interaction, we can make predictions of interaction conservation from our duplicate models and compare this to known interaction data from BioGrid. We used the MC network to assess how likely a conservative or divergent substitution were to produce conserved or divergent interactions, respectively. Only 14 out of 35 duplicates with conservative interface substitutions conserved the interactions. Conversely, 85 out of 105 duplicates with divergent interfaces had divergent interactions (F-score 0.4). Although we can see that our simplistic definition of interaction conservation based on atom contacts has some predictive power, we still identify many interactions that diverge when interface substitutions maintain atom contacts. One potential explanation is the presence of false negatives in our data but it is also likely that the maintenance of atom contacts is not the only factor affecting the conservation of interactions. In addition to considering the effect of substitutions on atom contacts in the interface, among others, the effects on structural integrity of the interface and solubility in the unbound state should also be taken into account. Ultimately, a wide range of factors may contribute to the evolution of interaction interfaces and their specificity.

## Conclusions

Physical interactions between proteins are essential for almost all biological functions and if we are to understand the evolution of function we need a complete understanding of how physical interactions change and evolve. We have shown that physical interactions are lost more rapidly after gene duplication than previously thought, indicating that interactions may regularly change. Building on previous work that demonstrated changes in interactions cannot be explained by changes along the length of the protein we have shown that changes in interactions are only loosely correlated with changes in interface regions. Consequently, we have found that many changes in interface regions can be equivalent with respect to binding and that neutral evolution is common in interfaces. Our results indicate a complex relationship between sequence, structure and function by showing that changes along a protein sequence and furthermore, identifying changes in interfaces, is not sufficient for predicting changes in interactions. Instead, we identify the need for a structural view of protein-protein interaction evolution that examines substitutions in structural context taking into account position, structural integrity of the interface, solubility in the unbound state and atoms that make contacts across the interface. Finally, given the propensity of functionally-equivalent changes in interfaces, we conclude that such changes may have an important evolutionary role by contributing to evolutionary plasticity in interfaces and creating cryptic variation, which in turn may provide the raw material for functional innovation and coevolution.

## Availability of data and materials

The whole-genome and small scale duplicates used in this study are available from [8] and [14] respectively. In addition these duplicates along with estimates of substitution rates are also available in Additional file 1: Table S1. Models of duplicate structures in complex, along with the atom contact images used to identify interaction interfaces, are available on request.

## Additional files

Additional file 1
**Table S1.** A list of known whole-genome and small-scale duplicates complete with estimates of substitution rate and codon usage bias used in this study. (XLSX 66 kb)

Additional file 2
**Figure S1.** The relationship between the interface substitution rate relative to non interface residues and the shared interaction ratio (SIR) between duplicates. SIR was calculated using multiple confidence interaction data (red points) and all interaction data (blue points) contained in BioGrid. A cutoff of 2 was used for the relative interface substitution rate to ensure reliable estimates. The red and blue lines represent the lines of best fit for the multiple confidence data and all interaction data respectively. (PDF 735 kb)

## Abbreviations

PPI: protein-protein interaction
DOPE: discrete optimised protein energy
SIR: shared interaction ratio
