# Supplementary figures and images for: Binding interface change and cryptic variation in the evolution of protein-protein interactions
Source: BMC Evol Biol. 2016 Feb 18;16:40. doi: 10.1186/s12862-016-0608-1 (PMC4758157; doi:10.1186/s12862-016-0608-1)

Supplementary Figure 1

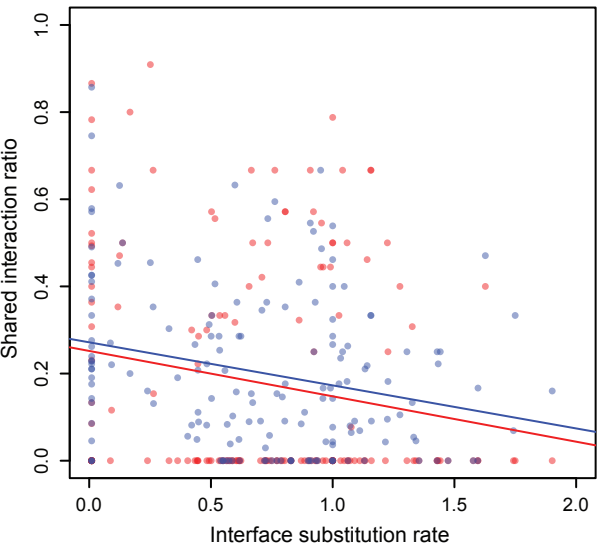

Supplement: Additional file 2 — Figure S1. The relationship between the interface substitution rate relative to non interface residues and the shared interaction ratio (SIR) between duplicates. SIR was calculated using multiple confidence interaction data (red points) and all interaction data (blue points) contained in BioGrid. A cutoff of 2 was used for the relative interface substitution rate to ensure reliable estimates. The red and blue lines represent the lines of best fit for the multiple confidence data and all interaction data respectively. (PDF 735 kb) [file 12862_2016_608_MOESM2_ESM.pdf]
